# Distinct Neural Signatures for Very Small and Very Large Numerosities

**DOI:** 10.3389/fnhum.2017.00021

**Published:** 2017-01-31

**Authors:** Michele Fornaciai, Joonkoo Park

**Affiliations:** ^1^Department of Psychological and Brain Sciences, University of MassachusettsAmherst, MA, USA; ^2^Commonwealth Honors College, University of MassachusettsAmherst, MA, USA

**Keywords:** event-related potentials, approximate number system, numerosity processing, subitizing, texture-density

## Abstract

Behavioral studies of numerical cognition have shown that perceptual threshold for numerosity discrimination depends on the range of numerical values to be estimated. Discrimination threshold is constant when comparing very small numerosities via the mechanism called subitizing, while it increases as a function of numerosity for numbers beyond that range governed by subitizing. However, when numerosity gets so large that the individual elements start to form a cluttered ensemble, discrimination threshold increases as a function of the square root of numerosity. These behavioral patterns suggest that our sense of number is not based on a unitary mechanism and is rather based on multiple numerosity processing mechanisms depending on the absolute numerosity to be estimated. In this study, we demonstrate neurophysiological evidence for such multiple mechanisms. Participants’ electroencephalogram (EEG) was recorded while they viewed arrays containing either very small (1–4) or very large (100–400) number of dots with systematic variations in non-numerical cues. A linear model that tested the effects of numerical and non-numerical cues on the visual-evoked potentials (VEPs) revealed strong neural sensitivity to numerosity around 160–180 ms over right occipito-parietal sites irrespective of the numerical range presented. In contrast, earlier neural responses (~100 ms) showed markedly distinct patterns across the different numerical ranges tested. These results indicate that differences in behavioral response patterns in numerosity estimation across various numerical ranges may arise from the differences in the first stages of visual analysis. Collectively, the findings provide a firmer ground for the idea that there exists a brain system specifically dedicated for numerosity processing, yet they also suggest that multiple early visual cortical mechanisms converge to that numerosity processing stage later in the visual stream.

## Introduction

While human linguistic abilities allow developing specific codes and symbols for numbers, which in turn lead to abstract numerical concepts and formal mathematics, we also possess a more intuitive and automatic ability: the ability to rapidly tell the approximate number of items in a given visual scene (or numerosity), without the opportunity to count them (Gallistel and Gelman, 1992; Dehaene, 2011). Such approximate estimation is indeed a more primitive capacity, already present in newborns (i.e., Xu and Spelke, 2000; Izard et al., 2009; Starr et al., 2013), shared with non-human primates (Hauser et al., 2000) and with a wide range of other vertebrates (Emmerton and Renner, 2006; Pepperberg, 2006; Agrillo, 2014). From an evolutionary point of view, this primitive ability gives important advantages for survival, since number represents one fundamental property of the environment, and it can be regarded as a *primary* perceptual feature (Burr and Ross, 2008; Stoianov and Zorzi, 2012; Arrighi et al., 2014; Anobile et al., 2015; Fornaciai et al., 2016).

The idea of a specific mechanism for the processing of approximate numerical magnitudes, however, has been highly debated in the past years. Indeed, according to some authors (Durgin, 1995, 2008; Durgin and Proffitt, 1996; Dakin et al., 2011), numerical information might be extracted by other mechanisms not primarily dedicated to number, and particularly by mechanisms specific for the processing of texture-density information. Nevertheless, an increasing amount of evidence both from psychophysical (i.e., Arrighi et al., 2014; Fornaciai et al., 2016) and neuroimaging studies (i.e., Harvey et al., 2013; Park et al., 2016) suggests that this is not the case, and that approximate numerical abilities are likely subserved by a dedicated neural circuitry.

Furthermore, according to recent frameworks (see Anobile et al., 2016 for a review) numerosity perception might be subserved by multiple mechanisms, preferentially engaged under different circumstances, and particularly as a function of the number of items to be estimated. The first of such multiple mechanisms reflects the rapid and accurate estimation of very small numbers (1–4)—a mechanism that has been named *subitizing* (Kaufman et al., 1949). The number of items in this range is immediately recognized, virtually without any error and no variability in estimates (Jevons, 1871/1911). Beyond four items, a second mechanism comes into play, representing more appropriately the action of the so-called approximate number system (ANS; Dehaene et al., 1999; Feigenson et al., 2004). When subjects are asked to discriminate two numerosities in the ANS range, the threshold (i.e., the just noticeable difference, JND) for a correct discrimination increases linearly as a function of numerosity, following Weber’s law (i.e., Whalen et al., 1999; Ross, 2003; Dehaene, 2011). However, when the number of items becomes so large that the individual elements start to be tightly packed, Weber’s law for numerical estimates is violated. Instead, discrimination threshold starts to increase with the *square root* of numerosity (Anobile et al., 2014). This third mechanism seems to draw information from different sources, particularly from texture-density analysis. It is interesting to note that the transition from numerosity to texture-density regime also depends on stimulus’ eccentricity with respect to the fovea (Anobile et al., 2015), resembling the dynamics of the “crowding” effect—a deleterious feature averaging that impedes objects segmentation and recognition (Levi, 2008; Pelli and Tillman, 2008). Particularly, Anobile et al. (2015) showed that while in central vision performances shift to the texture-density regime at a density of about 2.27 dots/degree^2^, at 5 degree of eccentricity the texture-density regime comes into play with just 1.19 dots/degree^2^. Thus, texture-density mechanisms might drive numerosity perception when the items become too cluttered to be individually recognized.

Regarding the neural underpinnings of approximate numerical magnitude processing, several studies investigated the idea that numerosity, particularly in the ANS range, is subserved by a specific mechanism independent of other visual attributes. For instance, many functional magnetic resonance imaging (fMRI) results converged toward indicating the human parietal cortex, and particularly the intraparietal sulcus (IPS), as the best candidate for the neural substrates of numerosity perception, showing a strong sensitivity to changes in numerosity (Piazza et al., 2004, 2007). Moreover, Harvey et al. (2013) further showed a topographically organized map for number in human parietal cortex, with populations of neurons tuned to different numbers, independently from other features, and organized in a graded fashion across the cortical surface.

More recently, Park et al. (2016) tested the temporal dynamics of neural activity underlying numerosity processing in the ANS range, with a novel technique allowing to test unique contributions of numerical and non-numerical attributes on neural responses (see also DeWind et al., 2015). In short, dot array stimuli were constructed systematically in equal ranges along three orthogonal dimensions of number (N), size (Sz) and spacing (Sp). This way, many visual properties of a dot array such as the total surface area (TA), individual dot area (IA), field area (FA), sparsity (Spar), coverage (Cov) and apparent closeness (AC) could be expressed as a linear combination of these three dimensions (see “Materials and Methods” Section). Using such a design, the authors analyzed the modulation along these different dimensions, and tested whether neural responses were more sensitive to changes in numerical magnitude, or to other visual properties. Their results demonstrated a strong sensitivity in the visual-evoked potentials (VEPs) primarily as a function of numerosity, while sensitivity to changes in other attributes was considerably weaker. This pattern of sensitivity started extremely early in the visual stream, as soon as 75 ms after stimulus onset over medial occipital sites, and continued at later latencies (180 ms) over bilateral occipito-parietal sites, suggesting the existence of a specific perceptual mechanism for processing numerosity information in a rapid and automatic fashion. Interestingly, these results seem to reflect the processing stages previously proposed in an influential model of numerosity perception (Dehaene and Changeux, 1993). Namely, while early activity (75 ms) might reflect the first stage of image *normalization*, which creates a size-invariant object location map, the later stage (180 ms) might reflect *summation* processes, where the output of the normalization stage is summed to represent numerosity.

**Figure 1:**
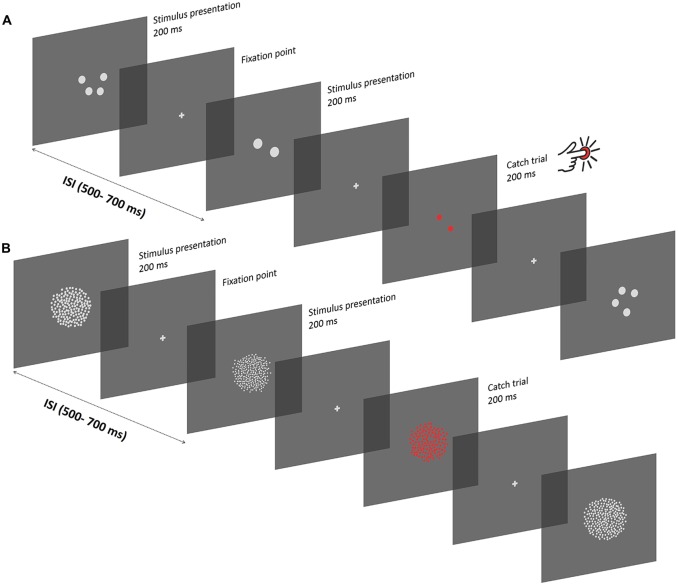
**Experimental procedure. (A)** Depiction of the experimental procedure in the subitizing condition. Stimuli were presented serially, and displayed for 200 ms, with a variable inter-stimulus interval of 500–700 ms. Between successive presentations, a gray fixation cross was displayed. Participants passively viewed the sequence of stimuli while keeping their gaze at the center of the screen. To ensure that participants pay attention to the screen, a stimulus composed of red items was displayed occasionally, and participants were instructed to press a button on a joypad as fast as they could (“catch” trials). In the subitizing condition, dot arrays comprised 1, 2 or 4 dots. Each stimulus was constructed in order to represent a unique combination of different numerical and non-numerical visual attributes, which can be represented as a linear combination of numerosity, size and spacing. **(B)** Texture-Density condition. Experimental procedures were the same as in the subitizing condition, except for the number of items in the stimuli (100, 200 or 400 dots) and for the specific values of other visual attributes (see “Apparatus and Stimuli” Section). Note that the stimuli are not represented in scale.

However, while these previous results demonstrate a unique neural signature showing specific sensitivity to numerosity in the ANS range, little is known about whether such a pattern generalizes to the perception of numerosities in different ranges of numerical values. Here, we aimed to test whether a similar sensitivity to numerosity is evident also in different ranges of numerosity, or whether other visual attributes contribute to neural responses. To this aim, we used the same design and analytic technique previously used in Park et al. (2016), but presenting stimuli comprising only very few dots (1–4)—i.e., numerosities processed by means of *subitizing* mechanisms—or comprising very large number of dots (100–400), i.e., in the *texture-density* range where dots form a tightly packed ensemble (see Figure 1). As in that previous study, stimuli were systematically constructed in order to cover similar ranges of several visual attributes, which can be represented as linear combinations of three orthogonal dimensions (N, Sz, Sp). If numerosity processing involves similar mechanisms throughout different numerical ranges, we would observe similar neural sensitivity patterns to numerosity and similar time courses as in the previous report (Park et al., 2016). Alternatively, if different (or at least partially different) mechanisms are involved in processing numerical magnitudes in different ranges, we would find different (or partially different) patterns of activation and sensitivity to numerical and non-numerical visual attributes.

## Materials and Methods

### Subjects

Thirty-three subjects (25 females, age range from 19 to 26 years) took part in the study after giving written informed consent, and were rewarded for their time with course credits. All the participants were naïve to the purpose of the study, and had normal or corrected-to-normal vision. Participants were screened for right-handedness (using Edinburgh Handedness Inventory) and for having no history of neurological, psychiatric and attentional disorders. Experimental procedures were approved by the University of Massachusetts (Amherst) Institutional Review Board and were in line with the Declaration of Helsinki.

### Apparatus and Stimuli

Stimuli were generated using the routines of the Psychophysics Toolbox (Brainard, 1997; Pelli, 1997; Kleiner et al., 2007), for Matlab (version r2013b; The Mathworks, Inc.), and presented on a monitor screen (ASUS VG248QE) encompassing approximately 34 × 19 degree of visual angle (from a viewing distance of about 90 cm), with a resolution of 1920 × 1080 pixels, and running at 144 Hz.

Stimuli were arrays of white dots presented on a black background. The arrays were systematically constructed to range equally in three orthogonal dimensions: numerosity (*N*), size (*Sz*) and spacing (*Sp*; see DeWind et al., 2015; Park et al., 2016). The two dimensions orthogonal to numerosity (i.e., Sz and Sp) were derived by logarithmically scaling and combining individual item area (IA), total area occupied by the items (TA), area of the circular field over which the dots were drawn (FA) and sparsity or the inverse of item density (Spar). Specifically, Sz represents the dimension along which both TA and IA change concurrently while N is held constant, and it is defined as log(Sz) = log(TA) + log(IA). On the other hand, Sp represents the dimension along which both FA and Spar are concurrently modulated while N is held constant, and it is defined as log(Sp) = log(FA) + log(Spar). In addition, two other non-numerical dimensions were defined based on *Sz* and *Sp*. The first one, apparent closeness (AC) represents the overall scaling of the dots independent of the number of items: increasing AC is equivalent to an increase in both Sz and Sp at the same rate, and is defined as log(AC) = $\frac{1}{2}$log(Sz) + $\frac{1}{2}$log(Sp). The second one, coverage (Cov), represents the total area (TA) divided by the FA, and it is defined as log(Cov) = $\frac{1}{2}$log(Sz) − $\frac{1}{2}$log(Sp). Importantly, all other non-numerical dimensions mentioned above (IA, TA, FA, Spar, AC, Cov) can be represented as a linear combination of the three orthogonal dimensions. The dot array stimuli were constructed so that each of the three dimensions consisted of three levels, which resulted in 27 possible stimulus types. However, in the subitizing condition, due to rounding error, there were 18 unique stimulus types instead of 27. Nevertheless, this rounding error did not affect the overall range of the dimensions spanned in Sz and Sp. Note also that the dimension of Sp is relatively meaningless for very small numerosities; therefore, that dimension is not considered during the analysis of the subitizing condition (see “Regression Analyses” Section below). For details of this stimulus construction scheme, see DeWind et al. (2015) and Park et al. (2016).

The parameters of the stimuli were set as follows: in the subitizing condition, the arrays contained 1, 2 or 4 dots. The minimum IA was set to 113.1 pixel^2^ (0.034 degree^2^), corresponding to a diameter of 0.1 degree (6 pixels), while the maximum IA was 452.4 pixel^2^ (0.13 degree^2^), corresponding to a diameter of 0.2 degree (12 pixel). The minimum FA was 3848 pixel^2^ (1.1 degree^2^), encompassing 0.6 degree of visual angle in diameter (70 pixels), while the maximum FA was 15,394 pixel^2^ (4.6 degree^2^), encompassing 2.4 degree in diameter (140 pixels). In the texture-density condition, the array contained 100, 200 or 400 dots. The minimum IA was 3 pixel^2^ (0.0009 degree^2^), corresponding to a diameter of 0.02 degree (1 pixels), while the maximum IA was 12.6 pixel^2^ (0.004 degree^2^), corresponding to a diameter of 0.07 degree (4 pixel). The minimum FA was 25,447 pixel^2^ (7.6 degree^2^), encompassing 1.5 degree of visual angle in diameter (180 pixels), while the maximum FA was 101,787 pixel^2^ (30 degree^2^), encompassing 6.2 degree in diameter (360 pixels). In all cases, the individual item area of the dots was homogeneous within each array and the minimum distance between any two dots was set to be no smaller than the radius of the dots. Regarding the Texture-Density condition, we chose the parameters of the stimuli also taking into account the less well-defined transition from the ANS to the texture-density regime (i.e., Anobile et al., 2014, 2015). Indeed, the combination of minimum N (100 dots) and maximum FA (30 degree^2^), gives rise to a density of 3.33 dots/degree^2^—which, according to previous studies (Anobile et al., 2015), should easily trigger the texture-density regime (i.e., even in central vision the critical density is about 2 dots/degree^2^).

### Task and Procedure

The experiment took place in a quiet and dimly illuminated room. Each participant first underwent the subitizing session that comprised two blocks of 400 trials, followed by the texture-density session that comprised six blocks of 400 trials. This difference in the number of blocks tested in the two conditions is due to the number of parameters analyzed in the different conditions. All three orthogonal dimensions (number, size and spacing) were used to characterize dot arrays in the texture-density condition, but only number and size were used to characterize dot arrays in the subitizing condition because spacing is virtually meaningless in that condition (see “Apparatus and Stimuli” Section). Thus, more trials were performed in the texture-density condition to account for more parameters. For the entire duration of the experiment, participants were instructed to keep their gaze at the center of the screen, which was signaled during the inter-stimulus interval by a gray fixation cross. Stimuli were randomly chosen from trial to trial, drawing from a set of 2700 pre-generated stimuli, and displayed for a duration of 200 ms, with a variable inter-stimulus interval (500–700 ms) between each presentation (Figure 1). The task involved participants to passively view the stream of dot patches, but in order to keep participants’ attention focused on the screen, a red stimulus was occasionally displayed among the others. In such cases participants were instructed to press a key on a joypad as fast as they could. Each block of trials contained 20 of those red oddball stimuli, with a variable occurrence among standard trials in order to avoid a regular and predictable presentation. Particularly, the distance between two consecutive oddball trials was randomly chosen, with values ranging from a minimum of 9 to a maximum of 19 standard trials between two consecutive oddballs. No other instructions were given to the participants. Prior to the actual experiment, participants performed a few practice trials until they saw at least two “catch” trials in order to ensure that they understood the task. Each block took about 5 min, and participants were free to rest between blocks. Overall, the total duration of the experiment ranged between 60 and 75 min.

Hit rates (M ± SD) in the oddball detection task were 95 ± 18% and 94 ± 18%, while the response times (M ± SD) were 411 ± 42 ms and 441 ± 50 ms, respectively for the subitizing and texture-density condition.

### Electrophysiological Recording and Data Analysis

Electroencephalogram (EEG) was recorded continuously for the entire duration of the experiment by means of a 64-channel, extended coverage, triangulated equidistance cap (M10, EasyCap, GmbH; actiCAmp, Brain Products, GmbH). EEG was recorded with a sampling frequency of 1000 Hz, and low-pass filtered at 100 Hz. All channels were initially referenced to the vertex (Cz) during recording. To monitor artifacts due to eye movements or blinks, the electro-oculogram (EOG) was monitored by means of electrodes positioned below the left eye and lateral to the left and right canthi. In rare cases, channel impedances up to 35 kΩ were tolerated, but they were kept below 15 kΩ in almost all the time.

Data were analyzed offline in Matlab (version R2013b), using the EEGLAB software package (Delorme and Makeig, 2004) and the associated ERPLAB toolbox (Lopez-Calderon and Luck, 2014). First, the EEG data was re-referenced to the average value of all 64 channels, and a high-pass filter (0.1 Hz) was applied. The continuous data were then segmented in 500-ms long epochs, time-locked to the onset of the stimulus (from −100 ms pre-stimulus to 400 ms after stimulus onset), with pre-stimulus interval baseline correction. To exclude trials containing eye-blink artifacts, we applied the step-like artifact rejection tool of ERPLAB, rejecting trials in which activity from the eye-channels exceeded a threshold of 30 μV (window width = 400 ms, window step = 20 ms). This led to an average rejection rate of 16.36% in the subitizing condition, and 19.12% in the texture-density condition. Finally, we selectively averaged the epochs for each of the 27 stimulus types, and low-pass filtered the data (30 Hz) prior to computing the grand average.

Average event-related potentials (ERPs) were then sorted along changes in the three orthogonal axes (N, Sz, Sp), and contrast waves (weights of +1 0 −1 for the three levels in each dimension) were computed for numerosity, size and spacing. The significance of the contrast waves was tested using a cluster-based non-parametric test with a height threshold of *p* < 0.001, using a simulation of a null distribution with 10,000 random sampling (Maris and Oostenveld, 2007). These analyses were, however, limited to the channels showing local peaks of the chi-square statistics obtained in the regression analysis (see “Regression Analyses” Section).

### Regression Analyses

A non-linear mixed effect model was used to assess the effects of experimental modulations along numerosity, size and spacing on the VEPs. For each channel, participants’ average ERP amplitude was calculated in several partially overlapping 50-ms windows, centered on latencies spanning over the entire trial duration (from −75 ms to 365 ms in 10 ms steps, for a total of 45 window centers), for all the 27 stimulus types. In a mixed-effects model, these average ERP amplitudes were entered as the response, and the three orthogonal regressors of N, Sz and Sp were entered as the fixed-effects with subjects as a random effect variable, allowing to take an additional (additive) random effect into account. Note that the dimension of spacing is less meaningful in the case of very small numerosities (e.g., the density of one or even two dots is underspecified). Thus, in the case of the subitizing condition, the mixed-effects model was run with two orthogonal regressors of numerosity and size by collapsing the dimension of spacing. However, to be sure that excluding spacing would not bias the results, we have also run the analysis including spacing in the model, finding an almost indistinguishable pattern of results. This confirms that when only few dots are presented their spacing is less relevant. For simplicity, we present and discuss only the results of the model without spacing in the subitizing condition.

**Figure 2:**
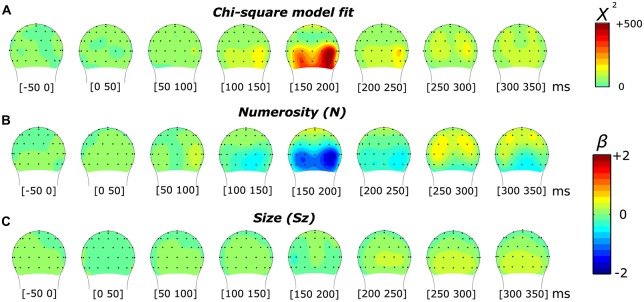
**Topographic distribution of the results of the non-linear mixed-effects model in the subitizing condition, which decomposes the event-related potential (ERP) modulation by numerosity and size. (A)** Chi-square model fit. **(B,C)** Beta estimates for numerosity **(B)** and size **(C)**.

In order to interpret the significance of the results provided by the model, we first compared the effects of the overall model (comprising one constant and three orthogonal regressors) against a null model (comprising only the constant) with a likelihood ratio test. The comparison between these two models resulted in a chi-square statistic for all 45 time points and across all the channels. Figures 2 and 6 (respectively for the subitizing and texture-density conditions) show the topographic distribution of the chi-square values, as well as the distribution of the fixed-effect parameter estimates of N, Sz and Sp (*β*_N_, *β*_Sz_ and *β*_Sp_).

Since the main question of this study was to assess the contribution of different visual properties in driving the neural response to numerosity in various ranges, we further analyzed the results of the model in order to determine which of the candidate properties (i.e., N, TA, IA, FA, etc.) best represents the direction of the parameter estimate vector $\vec{\beta }$ = (*β*_N_, *β*_Sz_, *β*_Sp_). In order to do so, we computed the angle between vector $\vec{\beta }$ and the dimensions for each other property for specific channels and latencies that appeared to reflect the largest effects of the overall model, as showed by the chi-square statistic peaks. Furthermore, to assess whether the significance of the angle difference between $\vec{\beta }$ and the axis closest to it, compared to the second closest dimension, we applied a bootstrapping approach to obtain two-tailed *p*-values. Namely, we generated a bootstrapping sample of $\vec{\beta }$ by running the mixed-effects model for a given channel and a given time point using a random sample of the participants (with replacement) for each repetition (with a total of 10,000 repetitions). Then, we compared the angle differences of the first and the second dimensions closest to $\vec{\beta }$, and the proportion of simulated samples where the angle of the closest dimension exceeded that of the second closest dimension was taken as the *p*-value indicating the significance of the angle difference between the two axes.

### Exploratory Analysis

While the previous analysis focused on the specific latencies and channels selected on the basis of the mixed-effects model output, we also explored neural sensitivity to numerical and non-numerical dimensions outside the pre-specified latencies and channels. To this end, we conducted an exploratory analysis testing whether $\vec{\beta }$ was “close” to any of the numerical and non-numerical dimensions in all time-windows (same as those in the regression analysis) in eight or ten posterior channels (respectively for the subitizing and texture-density condition; see Figures 5, 9). Specifically, we calculated the angle between the $\vec{\beta }$ vector and all other dimensions at each latency window and searched for latency points where the angle between $\vec{\beta }$ and any of the dimensions is smaller than the angle computed in the previous analysis based on the peak of the mixed-effects model output (final part of “Regression Analyses” Section). Besides comparing the angle at each time point with the angle obtained in the previous analyses, we also applied the additional constraints of having the norm of $\vec{\beta }$ at least equal to 50% of the same measure computed from the peak of the mixed-effects model output. In addition, we constrained the *p*-value of the model fit (obtained with the likelihood-ratio test, see “Regression Analyses” Section) not to exceed a Bonferroni-corrected threshold of 0.0055.

## Results

In the sections below, the results are discussed separately for the subitizing and texture-density conditions, following the same order of analyses reported in the “Materials and Methods” Sections (see “Regression analyses” and “Exploratory analysis” Section).

### Subitizing Condition

Figure 2 illustrates the results of a mixed-effects model quantifying the unique contribution of numerosity and size, as well as the overall goodness-of-fit in response to the dot arrays in the subitizing condition. The chi-square statistic showed a large local maximum at channel PO8’ (0.160 radians lateral to PO8 in the conventional 10-20 system, henceforth referred to as PO8’) at 165 ms. At PO8’, we also observed a smaller local maximum around 85 ms. Another peak was observed at channel O9’ (0.117 radians laterally to the position of O9 in the 10-20 system) around 165 ms. Both peaks in the overall chi-square statistics observed around 165 ms were associated with a clear negative-polarity modulation for the numerosity dimension (i.e., as showed by the beta values reported in Figure 2B). In contrast, the earlier (85 ms) chi-square peak at PO8’ was associated with a positive-polarity modulation in the numerosity dimension (Figure 2B). On the other hand, no apparent modulation was evident along the dimensions of size (beta values reported in Figure 2C).

**Figure 3:**
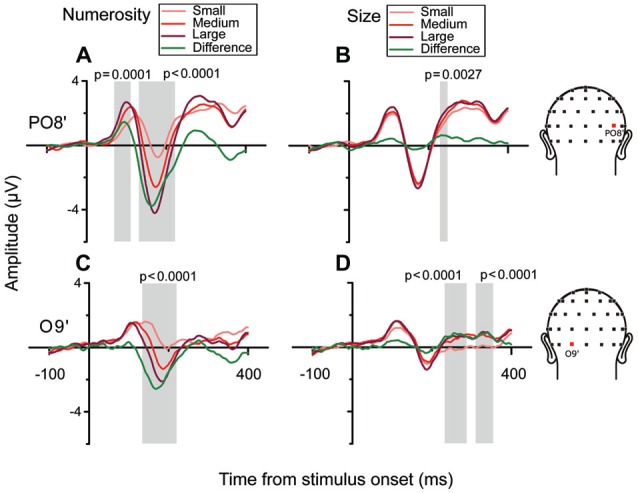
**Brainwaves from the right occipito-parietal channel PO8’ and the left occipital channel O9’ in the subitizing condition. (A,B)** From left to right, brainwaves sorted along changes in different dimensions: **(A)** N and **(B)** Sz for channel PO8’. **(A)** Responses to different numerical magnitudes. ERPs were sorted pooling trials in which different numerical magnitudes were presented, and depicted in increasing order from light red to darker red. The waveform depicted in green represents the linear contrast between the brainwaves relative to the highest and the lowest number. **(B)** Different magnitudes of Sz were sorted similarly to N. **(C,D)** Brainwave plots for channel O9’ (same conventions as **A,B**). Shaded areas and their respective *p*-values refer to the results of the cluster-based non-parametric test.

In order to better characterize the temporal progression of the modulation by numerosity and size, we examined the brainwaves from PO8’ and O9’ that showed local maximum points in the model-fit statistics (see Figure 2A). As shown in Figure 3, different numerosities resulted in different ERP magnitudes, showing a gradient from the lowest to the highest number (from lighter red to darker red) in both channels. Especially at later latencies (around 165 ms), the smallest number elicited the smaller absolute negative-polarity deflection, while increasing the number of items increased the negative amplitude of this deflection. This gradient was also evident in the green contrast wave (weights of +1 0 −1 to the three levels of numerosity) in Figure 3. A cluster-based non-parametric test (see “Materials and Methods” Section), resulted in two latency windows in PO8’ that showed a significant effect of numerosity: from 70 to 115 ms and from 130 to 220 ms. In O9’, one significant latency window was identified, ranging from 130 to 220 ms. The effect of size, on the other hand, was relatively weaker in later latencies (PO8’, 230–250 ms, *p* = 0.0027; O9’, 230–288 ms, *p* < 0.0001; O9’, 308–354 ms, *p* < 0.0001). This systematic modulation by numerosity—shown in both the regression model and by the ERPs sorted along different dimensions—clearly suggests that neural responses are highly sensitive to changes in the number of items presented, while sensitivity to changes in size appears to be weaker.

**Figure 4:**
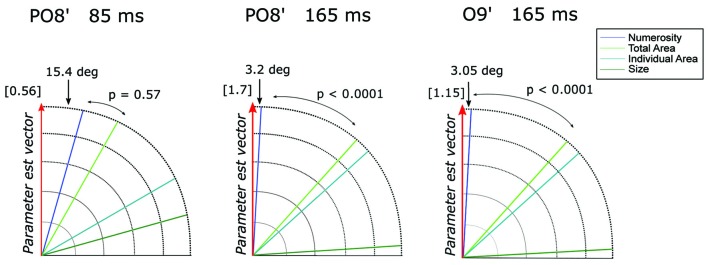
**The angle between each of the candidate dimensions and the parameter estimate vector at channel PO8’ around 85 ms and 165 ms and at channel O9’ around 165 ms.** *P*-values refer to the statistical significance of the comparison between the angle differences of the first vs. the second dimension closest to the parameter vector, obtained with a bootstrapping test. The norm of the parameter estimate vectors is reported in square brackets for each time window.

However, it should be noted that other non-numerical attributes often discussed in the literature (e.g., total surface area of the dots, surface area of an individual dot, area of the implicit circle in which the dots are draw, density of the array; see “Materials and Methods” Section) are represented by linear combinations of numerosity and size. Thus, to better quantify the contribution of these different visual attributes in the observed pattern of results, we evaluated which one of the candidate properties better represented the direction of the parameter estimate vector $\vec{\beta }$ = (*β*_N_, *β*_Sz_) at channel PO8’ around 85 ($\vec{\beta }$= [0.54, 0.15]) and 165 ms ($\vec{\beta }$ = [−1.70, −0.09]) and channel O9’ at 165 ms ($\vec{\beta }$ = [−1.14, −0.06]) (see “Materials and Methods” Section). Figure 4 shows the angle between $\vec{\beta }$ and various dimensions of the visual attributes. Numerosity resulted to be the dimension closest to $\vec{\beta }$ in all the three cases analyzed. Particularly, regarding the later latencies (165 ms), we found an angle difference of 3.2 degree and 3.05 degree, respectively for P08’ and O9’, followed by total area (TA) in both cases, respectively (angles = 41.8 degree and 41.9 degree). For both P08’ and O9’ at 165 ms, the angle difference between N and $\vec{\beta }$ resulted to be systematically smaller compared to that of the second closer axes (both *p* < 0.0001). However, the difference between N (angle = 15.4 degree) and TA (second closer dimension with an angle of 29.6 degree) axes at PO8’ around 85 ms did not reach statistical significance (*p* = 0.57). This result suggests that very small numerosities can explain significantly more variance in the observed ERPs compared to any other non-numerical dimensions of interest, but only at later latencies.

**Figure 5:**
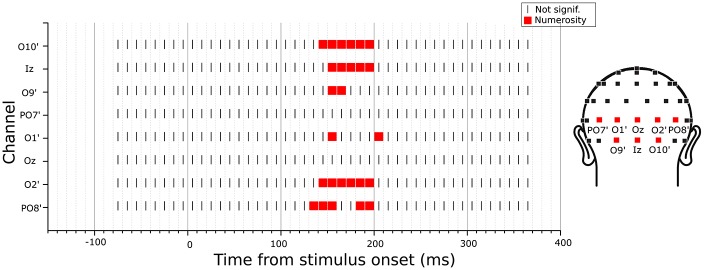
**Time-course of modulation in the posterior channels by the subitizing-range stimuli.** The dimension that was closest to the parameter estimate vector computed from the linear mixed-effects model under certain criteria (see “Exploratory Analysis” Section for details) was marked in each channel and latency point. The results revealed no other dimensions but numerosity to be modulating the neural activity.

Since the previous analysis was limited to specific channels and latencies, we searched for any potential VEP modulation by any numerical or non-numerical dimensions in different latency points during a trial. To do so, we looked for the angle between the parameter estimate vector and all other dimensions of interest, at each time point. Since in the previous analysis we found a maximum angle difference equal to 3.2 degree between $\vec{\beta }$ and N and a minimum $\vec{\beta }$ norm equal to 1.2, here we searched for angles equal or smaller than this value and with a norm at least equal to the 50% of 1.2. Figure 5 shows the results of this exploratory analysis for the eight posterior channels of O10’, Iz, O9’, PO7’, O1’, Oz, O2’, PO8’ (with O10’, O9’, O1’ and O2’ representing slightly lateral sites compared to the corresponding sites of O10, O9, O1 and O2 in the standard 10-20 system). Overall, only numerosity appeared to significantly modulate neural activity, and no other dimensions exceeded the threshold at any time point. Particularly, for all channels tested, numerosity appeared to have a consistent influence in time windows between 140 and 210 ms after stimulus onset.

### Texture-Density Condition

**Figure 6:**
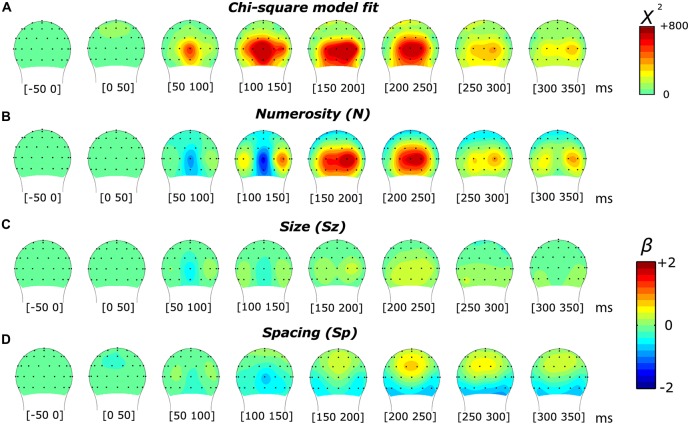
**Topographic distribution of the results of the non-linear mixed-effects model in the texture-density condition, which decomposes the ERP modulation by numerosity, size and spacing. (A)** Chi-square model fit. **(B–D)** Beta estimates for numerosity **(B)**, size **(C)** and spacing **(D)**.

As in the subitizing condition, we first analyzed the results of the texture-density condition using a linear mixed-effects model. As shown in Figure 6, we found a markedly different pattern of results compared to the subitizing condition (see Figure 2). In this condition, the chi-square statistic presented a more spread distribution along the temporal dimension, less lateralized compared to the subitizing condition. Particularly, we observed an early peak at around 105 ms, distributed onto midline occipital sites (Oz). At later latencies, the model-fit statistic peaked over O2’, at approximately 185 ms post-stimulus. The topographic distribution of beta estimates (Figures 6B–D), similarly to the subitizing condition, showed a modulation primarily by numerosity (Figure 6B), peaking at around 150–200 ms over O2’, while almost no sign of modulation was apparent along Sz and Sp (Figures 6C,D). Differently from the subitizing condition, the polarity of later responses was positive, with timing and scalp distribution compatible with the P2p component, previously reported to be involved with numerosity in the ANS range (Dehaene, 1996; Temple and Posner, 1998; Libertus et al., 2007; Hyde and Spelke, 2008; Hyde and Wood, 2011; Park et al., 2016).

**Figure 7:**
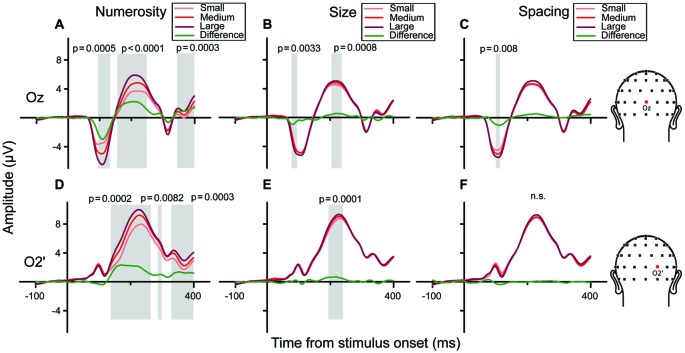
**Brainwaves for the central occipital channel Oz (A–C) and the right occipital channel O2’ **(D–F)** in the texture-density condition. **(A–C)**** Channel Oz: from left to right, brainwaves sorted along changes in different dimensions: **(A)** N, **(B)** Sz, **(C)** Sp. **(A)** Responses to different numerical magnitudes—ERPs were sorted pooling trials in which stimuli represented different numerical magnitudes, depicted in increasing order from light red to darker red. The green waveform represents the linear contrast between ERPs relative to the highest and the lowest number. **(B,C)** Different magnitudes of Sz and Sp were sorted similarly to N. **(D–F)** Brainwaves sorted along the different dimension for channel O2’, depicted in increasing order from light (lowest value) to darker red (highest value). Shaded areas and their respective *p*-values refer to the results of the cluster-based non-parametric test.

We then examined the brainwaves at the channels chosen as the local peaks of the regression model (Oz and O2’). Figures 7A–C show grand-averaged ERPs sorted along the three orthogonal dimensions of number, size and spacing (from left to right, respectively), for channel Oz. When ERPs were sorted to represent responses to different numerosities (panel A), a clear gradient of responses was evident (from lighter to darker red) with three peaks along the waveform. Particularly, analyzing the amplitude of the difference wave (showed in green) these three peaks corresponded to three statistically significant clusters of temporal windows: the first one spanning 93–133 ms (*p* = 0.0005), the second one spanning 154–250 ms (*p* < 0.0001) and a later one at about 340–390 ms (*p* = 0.0003). Regarding the right occipital channel O2’ (Figures 7D–F), the observed gradient of responses to different numbers was more pronounced at later latencies compared to Oz, with three significant peaks: the first ranging 136–264 ms (*p* = 0.0002), the second at 285–300 ms (*p* = 0.0082), and the last at 328–400 ms (*p* = 0.0003). These patterns were consistent with the regression model (Figure 6), showing a strong modulation of neural activity by N and little modulation by Sz and Sp.

**Figure 8:**
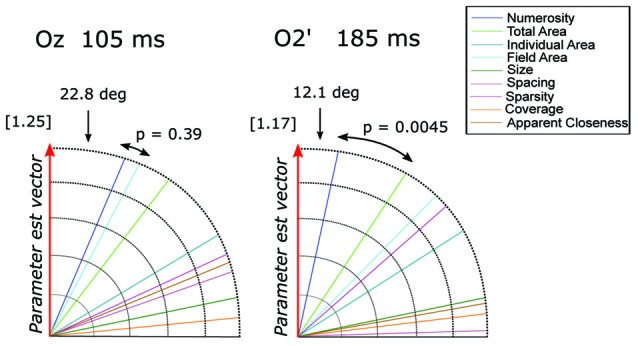
**The angle between each of the candidate dimensions and the parameter estimate vector at channels Oz and O2’.** *P*-values refer to the statistical significance of the comparison between the angle difference of the first closest dimension vs. the second dimension closest to the parameter vector. The norm of the parameter estimate vectors is reported in square brackets for each time window.

To quantify the contribution of different visual attributes in the observed pattern of ERP modulation, we tested which of the candidate dimensions best represents the direction of the parameter estimate vector $\vec{\beta }$ = (*β*_N_, *β*_Sz_, *β*_Sp_), for channels (Oz and O2’) at the respective peak latencies (105 and 185 ms; Figure 8). At Oz around 105 ms ($\vec{\beta }$ = [−1.14, −0.25, −0.41]), the dimension closest to $\vec{\beta }$ was N with the angle of 22.8 degree, followed very closely by field area (FA) with the angle of 27.5 degree. The difference between the two angles was not significant (*p* = 0.39). At O2’ around 185 ms ($\vec{\beta }$ = [1.18, 0.19, 0.0003]), numerosity was the closest dimension to the parameter estimates vector (12.1 degree) followed by total area (33.1 degree). In this case, the former was significantly smaller than the latter (*p* = 0.0045). These results suggest that while the modulation of neural responses to texture-density stimuli at later latencies (185 ms) is mostly explained by changes in numerosity, multiple visual attributes contribute to the variance of the earlier responses (105 ms).

**Figure 9:**
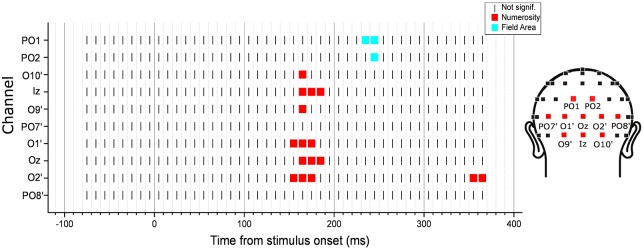
**Time-course of modulation in the posterior channels by the texture-density-range stimuli.** The dimension that was closest to the parameter estimate vector computed from the linear mixed-effects model under certain criteria (see “Exploratory Analysis” Section for details) was marked in each channel and latency point. The results revealed that neural activity was mostly modulated by numerosity, except for channels PO1 and PO2, where brain responses were modulated by field area (FA) at about 225–235 ms after stimulus onset.

We then performed an exploratory analysis searching for neural modulation by any of the dimensions of interest across the entire latency range in 10 posterior channels. Besides the eight channels tested in the subitizing condition, here we also added two other occipito-parietal channels (PO1 and PO2), which showed an effect of spacing in the regression analysis (see Figure 6). As in the subitizing condition, we computed the angle between the parameter estimate vector and the axes of all other dimensions, looking for cases where the angle is smaller than that observed for numerosity in the previous analysis (channel O2’, 12.1 degree), and with a norm of the $\vec{\beta }$ vector at least equal to 50% of $\vec{\beta }$ norm in the peak analysis (1.17). The results showed that ERPs were mostly modulated by numerosity, with the exception of channels PO1 and PO2, where brain responses appeared to be modulated by FA later in the time course (Figure 9). Moreover, differently from the subitizing condition, here we did not observe any specific and significant influence of numerosity at the two more lateral channels (PO7’ and PO8’), but only over more central sites. Activity at the other posterior channels showed a consistent modulation provided by numerosity, mostly at time windows comprised between 150 and 190 ms, with the exception of O2’, where we observed also a later modulation around 355–365 ms. On the other hand, FA modulated brain activity at about 225–235 ms after stimulus onset.

## Discussion

In the present study, we investigated the neural signature of numerosity processing in the subitizing and texture-density ranges. To this aim, we used the same experimental design previously developed by Park et al. (2016), which allows a systematic modulation of numerical and non-numerical visual attributes of the stimuli, that can be represented as variations along three orthogonal dimensions (numerosity, size and spacing). Participants viewed dot arrays with a very small (1–4) or very large number (100–400) of dots. Importantly, participants were not instructed to attend numerosity or any other magnitude feature of the stimuli, and numerosity itself was just one of the several visual attributes that varied from trial to trial. Thus, this stimulus design allowed an objective assessment of the unique effects of numerosity, size, spacing, and any combinations of those dimensions in explaining variations in the neural activity.

With numerosities processed by means of subitizing mechanisms, a strong sensitivity to numerosity was observed, while neural responses showed little if any sensitivity to non-numerical attributes. These results suggest that similarly to the ANS (see Park et al., 2016), numerosity processing in this range is largely unaffected by variations in other continuous, non-numerical visual properties. However, the modulation provided by changes in numerosity was apparent only at relatively later latencies (~165 ms) over the bilateral occipital sites. There were some signs of sensitivity to numerosity earlier in the time course (~85 ms, Figure 2B), but further analysis demonstrated that the sensitivity was not specific to numerosity (Figure 4). Moreover, such an early sensitivity was more apparent in the right occipital site (Figure 2B). These early ERP effects in the subitizing condition are in contrast to the previous report where ANS-range stimuli drove a strong ERP modulation by numerosity in medial occipital sites as early as 75 ms (Park et al., 2016). The topographic distribution of the later ERP effects are largely consistent with the distribution of the ERP effects driven by ANS-range stimuli (Park et al., 2016) except that the subitizing-range stimuli evoked negative polarity ERPs (Figure 2), which is in contrast to the ANS-range (Park et al., 2016) and the texture-density-range (Figure 6) stimuli that evoked positive polarity ERPs around 200 ms. This opposite polarity suggests that an entirely different part of the cortical surface may be differentially involved in processing very small numerosities. Particularly, according to Hyde and Spelke (2008), the negative-polarity modulation found with very small numbers would reflect a processing mechanism based on attentive object tracking, with amplitude differences reflecting the extent of parallel allocation of attention. On the other hand, the positive-polarity modulation at the level of the P2p component found with larger numerosities would reflect the processing of approximate numerical information independent from attention. Collectively, such a difference in the topographic distribution (in earlier latency) and the polarity (in later latency) suggests that anatomical locations responsible for processing very small numerosities are distinct from anatomical locations responsible for processing larger numerosities throughout the visual processing stream.

These negative-polarity peaks observed in the present study strongly resemble the VEP patterns found previously in studies using very small number of items (Libertus et al., 2007; Hyde and Spelke, 2008; Hyde and Wood, 2011), although previous studies mostly used paradigms involving sensory adaptation, where neural responses depend on the ratio between frequent and infrequent stimuli (Hyde and Spelke, 2008; Hyde and Wood, 2011). In our study, we focused on the neural responses elicited by the absolute numerical value of the stimuli, and our design allowed us to assess the contributions of other non-numerical visual attributes to the modulation of the ERPs and ruling out such visual attributes’ influence as cues for numerosity. Overall, our results seem fairly consistent with those previous reports, showing a negative-polarity deflection scaling with the numerical magnitude of the stimuli in the subitizing condition (Figure 3). As a novel addition to that body of knowledge, our data further provide evidence that neural activity is selectively modulated by numerosity, with little (if any) modulation by non-numerical visual attributes (e.g., size), providing a clear neural signature of the subitizing (parallel individuation) process.

In the texture-density condition, a clear sensitivity to numerosity was found especially in the later latency point (~180 ms), and this sensitivity to numerosity was again markedly stronger compared to the sensitivity to size and spacing. Here, differently from the subitizing condition, the polarity of this modulation was positive—that is, greater numerosity elicited greater amplitude of the positive-going ERPs. This positive deflection modulated by numerosity seems consistent with previous results concerning the ANS range (Dehaene, 1996; Temple and Posner, 1998; Libertus et al., 2007; Hyde and Spelke, 2008; Hyde and Wood, 2011; Park et al., 2016), which showed a systematic modulation of the P2p component—an ERP component thought to underlie the ANS. Indeed, our results showed a large effect of numerical magnitude over lateral (right) occipital sites (O2’), peaking at around 185 ms, which is consistent with the scalp distribution and timing of the P2p. At earlier latencies (~105 ms), we also found a moderate effect of changes in numerosity over midline occipital sites (Oz), although the ERPs were not selectively sensitive to numerosity. Overall, the topographic distribution and the timing of the modulation by numerosity, size and spacing in the texture-density condition are largely consistent with the previous results in the ANS-range stimuli (Park et al., 2016), suggesting similar anatomical regions may be responsible for the processing of both ANS-range stimuli and the texture-density range stimuli. Nevertheless, the weaker sensitivity to very large numerosities, compared to previous results on the ANS range (Park et al., 2016), suggests that different sources of information might concur in the formation of a numerosity representation at the early stages of visual processing. Interestingly, our exploratory analysis (Figure 9) also showed that information about field area are specifically represented later on during the course of activity (225–235 ms), but mostly over more superior channels (PO1 and PO2) compared to the effect of numerosity.

Regarding the topographical distribution of brain responses, our results show that relatively later activity peaks mostly in the right hemisphere. This preferential activation of the right hemisphere is indeed consistent with previous results (Piazza et al., 2004, 2007; Holloway et al., 2010; Park et al., 2013) suggesting that non-symbolic, approximate, numerical processing is predominantly carried out in the right hemisphere, and particularly in the right IPS, while symbolic or arithmetic tasks usually involve bilateral activity. Thus, our results suggest that this preferential activation of the right occipito-parietal sites for approximate numerosities extends also to different ranges such as very small numbers processed by means of subitizing mechanisms or very large numerosities in the texture-density range.

Overall, considering both ranges of numerosity tested in this study, our results are in line with Park et al. (2016), demonstrating a processing stage that shows a specific sensitivity to changes in numerosity especially at relatively later latencies (160–180 ms). Regarding the earlier latency effects, however, current results point out a major difference between ANS, subitizing and texture-density ranges. While a clear sensitivity to numerosity in the ANS range was found around 75 ms at the medial occipital site (Oz) in a previous study (Park et al., 2016), there was little evidence for such sensitivity to numerosity or to other magnitude dimensions in the other two ranges.

This lack of early responses in the subitizing condition might indicate the absence of a normalization stage. Indeed, such stage, needed to create a size-invariant object location map, could have been overcome by attentional parallel individuation processes. Much evidence indeed suggests a strong involvement of attention in the processing of small amounts of items. For instance, Ansari et al. (2007), investigating the difference between subitizing and ANS ranges, showed a differential involvement of attention-related areas, and particularly, of the temporo-parietal junction (TPJ)—an area that is known to be involved in controlling stimulus-driven (bottom-up) attention (Corbetta and Shulman, 2002). While TPJ showed an enhanced activation with subitizing stimuli, its activity was suppressed for stimuli requiring approximate estimation, supporting the idea that the extreme precision of numerical estimates concerning very small numerosities is supported by attentional resources. Similarly, Hyde and Wood (2011), measuring ERPs in response to small arrays of stimuli, found that when attention is overloaded by another task, neural responses no longer reflect modulation at the level of N1, but strongly resemble the typical patterns of responses usually measured with larger stimuli—that is, modulation at the level of the P2p component. Then, how may the attentional system support numerosity perception of very small numerosities? One possibility is that attentional engagement of object individuation operates in an “all or nothing” way: the attentional system could track one, two, three or even four items in parallel, and the same amount of attentional resources would be allocated irrespective of the number of items. Thus, early responses to subitizing stimuli would not show any relation to their numerosity, and the cardinality of the set of individuated items is extracted only at a later stage. Another possibility is that items processed by subitizing mechanisms could be bound together to form a set or a “chunk” of information to be stored in working memory, but maintaining access to the identity of individual objects (i.e., Feigenson, 2011). In this scenario, the items collected would be regarded as a single chunk, irrespective of their numerosity—which would be reflected by a relative insensitivity of neural responses to the number of objects collected at processing stages different from the extraction of the cardinal values itself.

In the texture-density range, the ERPs were sensitive to various magnitude dimensions over the medial occipital site early in the visual stream; however, the sensitivity was not specific to numerosity or to any other dimensions, unlike the case of the ANS range (Park et al., 2016). According to recent findings (Anobile et al., 2014, 2015), when the items become too cluttered and are subject to crowding, numerical estimates follow a different pattern of variability. Our results suggest that such regime change reflects changes at the early levels of visual processing, where different visual attributes might be combined to achieve an approximate representation of very large sets of stimuli. Indeed, while for subitizing stimuli a normalization stage may be no longer needed, with large ensembles it might not be even possible. Since cluttered (crowded) stimuli could no longer be segmented as separate objects, creating an object location map would be impossible. Thus, the visual system might rely on a different mechanism, like analyzing density or field area information to provide an approximate estimate of numerosity. Indeed, when objects are so cluttered that they form a uniform texture, information such as the area covered by the stimulus becomes surely more useful to represent it. While creating a normalized object location map could be the best way to avoid misleading influence on perceived numerosity in the ANS range, integrating several visual attributes might lead to a reduction of noise and uncertainty associated with magnitude representation in the case of cluttered stimuli (e.g., see Cheng et al., 2007 in the case of integration of spatial cues and Ernst and Banks, 2002 in the case of multisensory integration). Such a process seems reflected by the typical pattern of behavioral results obtained using texture-density stimuli, wherein thresholds for numerosity discrimination with cluttered stimuli depart from Weber’s law, and start to increase with the square-root of numerosity.

Interestingly, in the texture-density condition, the lack of specificity of early neural responses to any of the tested dimensions, on the one hand, could potentially suggest that such a unique stimulus condition elicits interference of numerosity representation by other visual cues. On the other hand, it could potentially suggest that different visual cues are integrated in that processing stage for a later representation of numerosity. This idea resembles an alternative account of numerosity perception, namely the sensory-integration account (e.g., Gebuis et al., 2016), which asserts that numerosity representation arises from the integration of various non-numerical, continuous cues. It is premature, however, to make a direct link between the current results and the sensory-integration account because there is no evidence yet to argue that the later selective sensitivity to numerosity (~185 ms) is directly driven by the early stages of magnitude processing (~105 ms) in the texture-density range. Indeed, in the subitizing condition, selective sensitivity to numerosity emerged around 165 ms but without any apparent earlier stage of magnitude processing, which suggests that the early effects of overall magnitudes in the texture-density range may not be a computational prerequisite to the later selective sensitivity to numerosity. Moreover, our set of results demonstrates that numerosity is the single dimension that is automatically and most robustly processed across various numerical ranges in the *absence* of an explicit magnitude task. Such results are difficult to be fully explained by the sensory-integration account, unless that account explains why various non-numerical visual cues would be integrated to form a representation of numerosity when there is no explicit need for representing number. In addition, such a non-specific early effect of magnitude was not observed for numerosities in the ANS range (Park et al., 2016), a finding that makes it difficult to generalize the sensory-integration account to all aspects of numerosity perception.

Finally, despite the differences in the early neural patterns evoked by dot arrays in different ranges of numerical values, later activity continues to be strongly sensitive to numerosity. These results suggest that irrespective of the initial processing stage (potentially carried out by attentional individuation, normalized object location map or texture-density processing), the information converges toward a later stage specifically dedicated to numerosity processing. The concept of an accumulator, or summation process, is proposed to be the core of the number system (Meck and Church, 1983; Dehaene and Changeux, 1993; Verguts and Fias, 2004). Our data suggest that this high-level mechanism might play a general role in numerosity perception, summing up information arising from earlier levels of analysis irrespective of the their exact nature, to achieve a more or less precise representation of the number of items. The difference at the early stages of neural processing highlighted in our study could also explain the systematic difference in behavioral performance when participants are asked to make numerical estimations in various ranges of numerical values. Particularly, different levels of noise arising from the initial stage of sensory analysis could determine the level of precision of numerical estimates at the later common accumulation stage. Indeed, it has been shown that the precision in behavioral tasks is strongly related to the level of noise in the output of sensory processing, for example in the case of temporal audio-visual processing (Hartcher-O’Brien et al., 2014).

In this context, the attentional individuation mechanism (subitizing) could only have a very limited amount of noise, since each item would be encoded separately as an individual object, leaving no uncertainty about the number of such few objects. In the ANS domain, the normalized object location map could provide a measure more resistant to biases from other visual attributes, but at the cost of a fairly high amount of noise, linearly incremental with the number of objects (Weber’s law). Finally, in the texture-density domain, where the first stage of processing could no longer be based on normalized objects, integrating different sources of information (i.e., number, density, area, size) could overall reduce the level of noise in the output of the first level of sensory processing, but potentially at the cost of a biased output. In other words, while in the ANS domain accuracy might be enhanced with the side effect of lower precision, in the texture-density domain precision might be enhanced by drawing information from sources that may bias the final estimate. However, crucially, such differences might arise only as a result of early processing, while numerosity information eventually converges toward a common accumulation mechanism.

## Conclusion

To conclude, our results provide new evidence concerning the neurophysiological underpinnings of numerosity perception in different ranges of numerical values. Using ERPs, we first showed that neural responses to dot arrays are strongly sensitive to changes in the absolute numerical magnitude of the stimuli especially at a relatively later latency, while sensitivity for other continuous visual properties is much more restrained. However, neural responses to subitizing stimuli showed a lack of sensitivity to numerosity and to any other magnitude dimensions at early latencies, suggesting the possibility to track each object by means of attention. Similarly, neural responses to texture-density stimuli showed only a weak sensitivity to numerosity at earlier latencies, suggesting that early responses to cluttered stimuli reflect an integration of numerical and non-numerical cues, since creating a normalized object location map with crowded stimuli could be no longer possible. Overall, the results provide evidence for the existence of multiple mechanisms of numerosity perception in different numerical ranges, and they suggest that differences in behavioral performances observed across different numerical ranges arise from differences in the perceptual processing mechanism very early in the visual stream.

## Author Contributions

JP conceived and conducted the experiment. MF and JP analyzed the data, interpreted the results and wrote the manuscript.

## Conflict of Interest Statement

The authors declare that the research was conducted in the absence of any commercial or financial relationships that could be construed as a potential conflict of interest. The reviewer MS and handling Editor declared their shared affiliation, and the handling Editor states that the process nevertheless met the standards of a fair and objective review.
